# Notable Differences in Clinical Features and Inflammatory Gene Expression Between Genital Lichen Sclerosus and Lichen Planus

**DOI:** 10.3390/biomedicines13112817

**Published:** 2025-11-19

**Authors:** Patrick Poffet, Veronika Baghin, Fabienne Fröhlich, Irina Banzola, Julia Laube, Mark Mellett, Lucie Heinzerling, Barbara Meier-Schiesser

**Affiliations:** 1 Department of Dermatology, University Hospital Zurich, 8091 Zurich, Switzerland; patrick.poffet@usz.ch (P.P.); veronika.baghin@usz.ch (V.B.); fabienne.froehlich@usz.ch (F.F.); julia.laube@usz.ch (J.L.); mark.mellet@usz.ch (M.M.); 2 Department of Urology, University Hospital Zurich, 8091 Zurich, Switzerland; irina.banzola@usz.ch; 3 Department of Dermatology & Allergology, LMU Klinikum, 80377 Munich, Germany; lucie.heinzerling@med.uni-muenchen.de

**Keywords:** lichen sclerosus, genital lichen planus, gene expression profiling, inflammatory mediators, inflammatory skin diseases

## Abstract

**Background/Objectives**: Genital lichen sclerosus (LS) and lichen planus genitalis (Lpg) are chronic inflammatory dermatoses with overlapping clinical features but incompletely understood pathogenesis. Current therapies are largely symptomatic. **Methods**: To clarify underlying mechanisms and identify therapeutic targets, we retrospectively analyzed 174 patients (142 LS and 32 Lpg). Clinical features and comorbidities were compared, and gene expression profiling of 730 inflammation-related genes was performed on lesional tissue from LS and Lpg patients and healthy controls using NanoString technology. Selected findings were validated by immunohistochemistry. **Results**: LS patients were older and predominantly female and more frequently had metabolic syndrome. On the molecular level, LS showed a generally lower inflammatory gene expression profile than Lpg. Nevertheless, LS was characterized by strong upregulation of CCL27 and MARCO, whereas Lpg displayed enhanced IL-1 pathway activation and increased expression of B-cell–associated markers. **Conclusions**: These results demonstrate distinct immunological differences between the two conditions and provide further insight into disease-specific pathways.

## 1. Introduction

Lichen sclerosus (LS) is a chronic, immune-mediated inflammatory dermatosis that predominantly affects females, particularly postmenopausal women and prepubertal girls. The condition exhibits a strong female predilection, with reported female-to-male ratios ranging from approximately 3:1 to 10:1 in adults. Prevalence estimates in postmenopausal women reach up to 3%, while in prepubertal girls it is approximately 0.1%. Although less commonly diagnosed in males, LS also occurs in both adult and pediatric male populations, with an estimated prevalence of up to 0.5% in boys and below 0.1% in adult males, considering that these data are primarily derived from studies conducted in Caucasian populations [1,2]. The disease typically manifests in the anogenital region. Its acute stage is characterized by severe itch, bruising, fissures, and pain, while the chronic stage presents with white, shiny, porcelain-like patches, leading to scarring and labial fusion with stenosis of vaginal introitus and urethral ostium at its most advanced stage. Extragenital manifestations are common [3]. Histologically, LS is characterized by an atrophy of the epidermis with follicular hyperkeratosis, atrophy of the elastic fibers and edematous impregnation of the hyalinized collagenous fibers in the upper dermis, and a band-like lymphocytic infiltrate in the lower dermis [4]. Currently, there is only symptomatic treatment available, mostly with topical high-potent glucocorticoids (GCs), whereas male patients should undergo circumcision [5]. However, a progress of the disease is still possible, which can lead to severe pain, itching, dysuria, and sexual inactivity [6]. The lack of targeted therapies is the result of a missing in-depth understanding of the disease’s pathomechanisms. Previous studies suggest a T-cell-mediated inflammatory dysregulation, where a Th1 IFN-γ-induced immune response plays a major role in the disease development [7]. However, the exact pathomechanism of the disease development remains unclear.

LS shows a lot of similarities to the genital form of lichen planus (LP), an inflammatory dermatosis of unknown origin in which about 25% of patients have genital involvement (lichen planus genitalis; Lpg) Both conditions can present with pruritus, burning, pain, white atrophic or erosive lesions, architectural distortion, and a chronic relapsing course, often leading to scarring and an increased risk of malignant transformation. Histologically, compared with LS, where the band-like lymphocytic infiltrate is localized in the lower dermis under a homogenized area of collagen, in Lpg the lymphocytic infiltrate lies directly beneath the epidermis. Nevertheless, in early stages, both diseases may show histological overlap as they both show a lichenoid/interface dermatitis with basal cell damage and a band-like lymphocytic infiltrate, making distinction difficult. As lesions evolve, LS typically develops papillary dermal hyalinization, loss of elastic fibers, and basement membrane thickening, whereas Lpg shows wedge-shaped hypergranulosis, saw-tooth acanthosis, and numerous Civatte bodies. Clinicopathologic correlation and, when necessary, repeat biopsy are often required for accurate diagnosis [2,7,8]. Like in LS, only symptomatic treatment is yet available with a combination of a topical GC and a topical calcineurin inhibitor, which are often unable to stop the further progression of the disease, highlighting the urgent need for a targeted therapy [9]. Various studies have shown that in Lpg, which is considered to be an immune-mediated disease, autoreactive T cells play an important role through dysfunctional cytokine production, particularly IFN-γ [10,11].

In summary, LS and Lpg share many similarities, both clinically and histologically, as well as in terms of availability and limitations of treatments. However, despite these overlaps, direct molecular comparisons between the two conditions are limited, and their distinct inflammatory profiles remain poorly characterized. This knowledge gap hinders the development of effective, targeted therapeutic approaches. For this purpose, data from patients of the University Hospital of Zurich, collected over a period of ten years, were analyzed. To identify specific cell populations and inflammatory mediators involved in LS, we performed a genetic analysis using NanoString and compared them with Lpg and healthy individuals from newly taken tissue biopsies. Additionally, we performed immunohistochemical staining of selected highly expressed genes to confirm our findings.

## 2. Materials and Methods

This study was conducted as a retrospective observational cohort study, analyzing clinical, histological, and molecular data from patients diagnosed with LS or Lpg.

Patient data (*n* = 174) from databases of the dermatology department of the University Hospital of Zürich, collected over the past 10 years, were retrospectively analyzed. From the 174 patients, 142 were suffering from LS and 32 from Lpg. Lesional skin/mucosa biopsies (gene expression analysis, *n* = 4; immunohistochemical analysis, *n* = 7) of patients suffering from each LS and LPG and non-diseased controls (NDCs; gene expression analysis, *n* = 3; immunohistochemical analysis, *n* = 5) from healthy individuals were examined. The diagnoses from the databases resulted from patient examination based on medical history, clinical evaluation, and histology.

Clinically, LS was characterized by porcelain-white, atrophic plaques with a sclerotic surface and frequent erosions or fissures, typically involving the anogenital skin. Histopathological findings required for inclusion comprised thinning of the epidermis with hyperkeratosis; loss of rete ridges; basal cell vacuolization; and a homogenized, sclerotic zone of collagen in the upper dermis, accompanied by a band-like lymphocytic infiltrate.

Lpg cases were defined by characteristic clinical and histopathological features of erosive or papular genital lichen planus. Clinically, patients presented with erosions, glazed erythematous patches, or violaceous papules, often accompanied by burning, pain, or dyspareunia. Histopathological confirmation required the presence of a band-like lymphocytic infiltrate at the dermoepidermal junction, basal cell degeneration, Civatte bodies, and saw-tooth acanthosis, in the absence of dermal sclerosis typical of LS.

Only patients with complete clinical documentation, fitting histopathology, and no overlapping or mixed features of LS and Lpg were included. Cases with uncertain diagnosis or insufficient data were excluded after multidisciplinary consensus review by two clinicians with expertise in genital dermatoses and a dermatopathologist.

This study was approved by the local ethics committee of Zurich (KEK 2021-00958) and the ethics committees of the University Hospital Erlangen (Nr. 2_20 B, Nr. 17_16Bc) and of the Ludwig-Maximilians-University, Munich (Project-nr. 20-1122, with amendment from 9 February and 21 February 2022). All human samples were taken after informed written patient consent and according to the principles of Helsinki.

All patients were systematically evaluated for relevant comorbidities, with particular attention to the presence of metabolic syndrome. For the purposes of this study, metabolic syndrome was defined as the coexistence of two or more of the following conditions: hypertension, obesity, type 2 diabetes mellitus, or dyslipoproteinemia. Additionally, patients were screened for autoimmune diseases. The following autoimmune conditions were identified within the cohort: Hashimoto’s thyroiditis, rheumatoid arthritis, vitiligo, alopecia areata, psoriasis vulgaris, and Graves’ disease.

For gene expression and immunohistochemical comparisons, a cross-sectional sampling strategy was used, selecting lesional and non-lesional biopsies based on diagnostic confirmation and RNA/tissue quality. Biopsies were taken from lesional skin/mucosa of LS and Lpg patients, as well as from non-diseased skin and mucosa from NDCs. In all three groups, the biopsy sites included both cutaneous and mucosal areas. For both gene expression and immunohistochemical analysis, the skin/mucosa was formalin fixed (4%) and paraffin embedded (FFPE).

Paraffin-embedded skin sections from 5 LS, 5 Lpg and 3NDCs were included. Immunohistochemistry was performed using antibodies targeting MARCO (rabbit anti-MARCO, 1:750, Invitrogen, Carlsbad, CA, USA), CCL27 (mouse anti-CCL27, 1:33, clone 124308, invitrogen, Carlsbad, CA, USA), and CD20 (mouse anti-CD20, 1:100, clone L26, Roche, Basel, Switzerland). Paraffin-embedded skin sections underwent deparaffinization and rehydration. To expose antigens, the slides were subjected to 25 min of heating using Target Retrieval solution (DAKO, Glostrup, Denmark). Following a one-hour blockage with 5% BSA in PBS, the sections were incubated with primary antibodies. The latter were identified using a biotinylated secondary antibody (1:100, SouthernBiotech, Birmingham, AL, USA), and positivity was visualized by an AEC chromogen substrate (Abcam, Cambridge, UK). Hematoxylin solution was used to counterstain. After fixation with DAKO mounting medium, the slides were scanned using ScanScope (Leica Biosystem, Vista, CA, USA), and semi-quantification was performed by a pathology-trained dermatologist using the Aperio ImageScope software (version 12.4.6, Leica Biosystem, Vista, CA, USA).

RNA was isolated using Trizol and the RNeasy FFPE Kit (Qiagen, Germantown, MD, USA) according to the manufacturer’s instructions. The gene expression analysis for 730 inflammation-related genes was performed using NanoString. The nCounter PanCancer Immune Profiling PanelTM (human) (Nanostring, XT-CSO-HIP1-12, Seattle, WA, USA) was used to determine expression. Sample hybridization was performed according to the manufacturer’s protocol after application of 200–400 ng of RNA. For sample detection and analysis the nCounter^®^ Flex Digital (Seattle, WA, USA) analyzer was used.

Initial data processing, quality assessment, and normalization were conducted using ROSALIND^®^ analysis software (v3.35.3.0; San Diego, CA, USA; https://rosalind.onramp.bio/, accessed on 1 April 2024) following established protocols [12]. Parallel analysis was also performed using nSolver^®^ software (v4.0, NanoString Technologies, Seattle, WA, USA).

Statistical analyses were performed using a combination of validated web-based tools. A two-tailed significance level of *p* < 0.05 was applied throughout. Categorical variables, such as sex distribution, the presence of metabolic syndrome, and autoimmune comorbidities, were compared between groups using the Chi-squared test for independence (Social Science Statistics, https://www.socscistatistics.com/tests/chisquare2/default2.aspx, accessed on 15 January 2025). Continuous variables, including age at diagnosis, were analyzed using the non-parametric Mann–Whitney U test, which is suitable for skewed distributions (StatsKingdom, https://www.statskingdom.com/170median_mann_whitney.html, accessed on 15 January 2025). To assess differences between observed and expected proportions, such as the prevalence of metabolic syndrome in LS patients compared with the general population, a one-sample proportion Z-test was used (MedCalc, https://www.medcalc.org/calc/test_one_proportion.php, accessed on 15 January 2025). Measures of variability, including 95% confidence intervals for proportions and interquartile ranges for age, were computed using Python (version 3.13.2) and NumPy (version: version 2.1.0).

For gene expression analysis, fold changes and *p* values were calculated using the fast method, described in the nCounter^®^ Advanced Analysis 2.0 User Manual (nCounter Advanced Analysis 2.0 Plugin for nSolver Software User Manual). *p*-values were adjusted using the Benjamini–Hochberg method of estimating false discovery rates. As cutoffs, a *p*-value adjustment of ≤0.05 and a fold change of 1.5 were defined for the comparison between LS and Lpg and a cutoff of ≤0.01 *p*. adj. and a fold change of two for comparison between LS and NDC.

Enrichr for Gene Ontology (GO) and KEGG 2021 Human was used for functional enrichment analysis and pathway analysis of significant genes [13]. Several databases such as Interpro, NCBI, REACTOME, and WikiPathways were referenced for enrichment analysis. A *p*-value adjustment ≤ 0.05 (≤0.01 in LS comparison to Lpg) was set as the cutoff for significance. For further information of significant genes, UniProt and The Human Protein Atlas were used [13,14,15].

## 3. Results

### 3.1. Clinical Patterns of LS and Lpg Are Distinct Across Age, Sex, Localization, and Comorbidities

A total of 142 patients with lichen sclerosus (LS) were analyzed over a ten-year period. The median age at diagnosis was 62 years, with a wide age range from 14 to 93 years. In contrast to previously reported data, no increased incidence was observed in prepubertal females in our cohort. The Lpg cohort comprised 32 patients, with a median diagnosis age of 53.5 years, ranging from 20 to 78 years. When comparing LS with Lpg, LS patients were generally diagnosed at an older age (*p* = 0.004) (Table 1).

The LS group consisted predominantly of females (*n* = 78; 55%). Conversely, the Lpg group had a higher proportion of male patients (*n* = 22; 69%). This distribution suggests a stronger male predominance in our Lpg cohort compared with LS, which was statistically significant (*p* = 0.016) (Table 1).

Regarding lesion localization, in LS, 88% of LS cases (*n* = 125) presented with genitoanal involvement, while the remaining 12% (*n* = 17) had extragenital manifestations, primarily affecting the shoulder, mammary, and abdominal regions. In the Lpg cohort, 53% (*n* = 17) reported only genital involvement, whereas 47% (*n* = 15) exhibited both genital and extragenital manifestations. Collectively, Lpg patients were more likely to experience extragenital manifestations compared with LS patients, with this difference being highly significant (*p* = 0.00001) (Table 1).

Additionally, patient cohorts were examined for comorbidities. Notably, LS patients had a significantly higher occurrence of metabolic syndrome-related diseases compared with those with Lpg (LS, 50%; Lpg, 22%; *p* = 0.039). In comparison, the prevalence of these conditions in the general population is 20.9% [16]. In particular, the prevalence of type 2 diabetes mellitus was higher in LS patients (LS, 13%; Lpg, 3%; *p* = 0.6), although this difference did not reach statistical significance. Notably, the prevalence in LS patients exceeded that of the general population (8.5%) [17]. Autoimmune diseases including Hashimoto’s diseases, rheumatoid arthritis, vitiligo, alopecia areata, psoriasis vulgaris, and M. Basedow were equally present in LS and Lpg patients (LS, 31%; Lpg, 28%; *p* = 0.676). In comparison, studies estimate that only 7.6–9.4% of the global population are affected by one or more autoimmune diseases [18]. Additionally, when comparing HBV or HCV infections in our cohort, no significant difference was detected between the two groups (LS, 5%; Lpg, 7%; *p* = 0.76). In the general population, the estimated prevalence of chronic HBV infection ranges from 3.2% to 3.8% globally and approximately 0.3% to 0.7% in Switzerland, while chronic HCV infection affects about 1% of the global population and less than 0.1% in Switzerland. Therefore, an increased prevalence was noticed in our study cohort.

### 3.2. Inflammation-Related Genes Are Lower Expressed in LS Compared with Lpg

**Figure 1:**
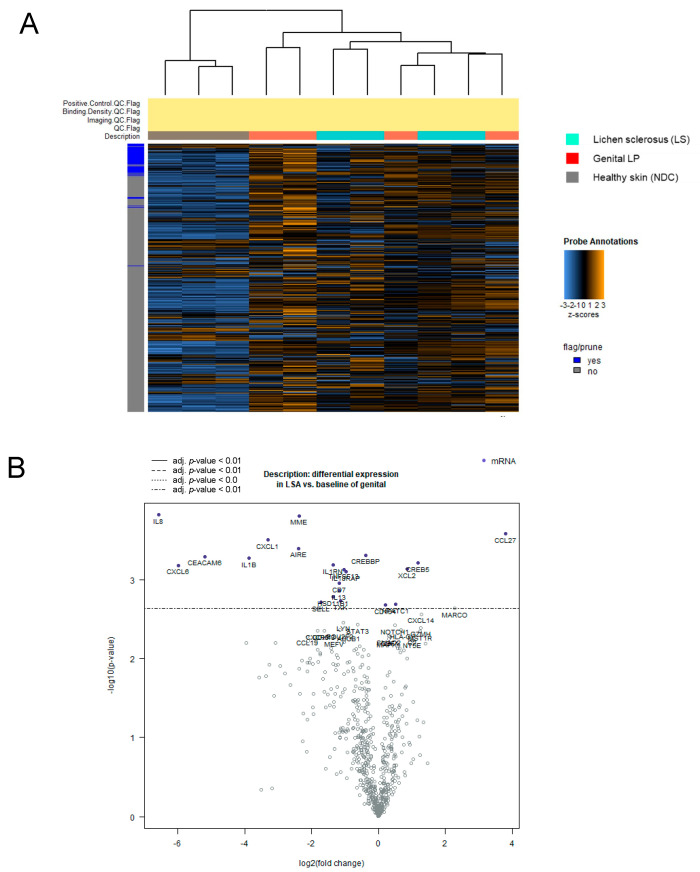
Molecular separation of lichen sclerosus, genital lichen planus, and healthy controls by gene expression profiling. NanoString technology was applied to compare the expression of 730 inflammation-related genes across lesional skin samples from Lpg (*n* = 4), LS (*n* = 4), and NDC (*n* = 3) samples. Heatmap visualization shows hierarchical clustering of genes in Lpg, LS, and NDC samples (**A**). Comparative analysis between LS and Lpg specimens identified 104 differentially expressed genes, with a predominance of upregulated genes in Lpg (86 genes) vs. LS (18 genes) (**B**). LS, lichen sclerosus; Lpg, lichen planus genitalis; NDC, non-diseased controls.

To explore the transcriptomic landscape of LS in comparison with Lpg and NDC, we conducted a comprehensive NanoString analysis of diseased (LS, *n* = 4; Lpg, *n* = 4) and healthy skin (*n* = 3), comparing the gene expression of 730 genes related to inflammation and cancer. Heatmap visualization revealed that LS and Lpg lesional samples clustered together, indicating comparable molecular signatures, whereas non-lesional skin samples formed a separate, clearly distinct cluster (Figure 1A). Comparing LS with NDC, we found 182 differentially expressed genes, where 175 genes were upregulated, whereas only seven were downregulated (Supplementary Tables S1 and S2) (*p* ≤ 0.01, log2 cut-off 1.5-fold). The predominantly upregulated genes were linked to cytotoxicity, NK cell functions, antigen processing, T-cell-mediated immunity, and pathogen defense mechanisms (Supplementary Table S3). In a parallel comparison between LS and Lpg specimens, 104 genes with altered expression levels were identified, predominantly skewed toward upregulated (86 genes) in Lpg compared with LS, while a minority (18 genes) showed upregulation in LS compared with Lpg (Figure 1B; Table 2 and Supplementary Table S4) (*p* ≤ 0.05, log2 cut-off 1.5-fold). Gene Set Analysis Significance Scores were only slightly different between LS and Lpg. Notably, the cytotoxicity and complement score was higher in LS compared with Lpg, while genes associated with Toll-like receptors (TLR), TNF superfamily and chemokines were higher in Lpg (Table 3).

### 3.3. CCL27 and MARCO Are Strongly Upregulated in LS Compared with Lpg

**Figure 2:**
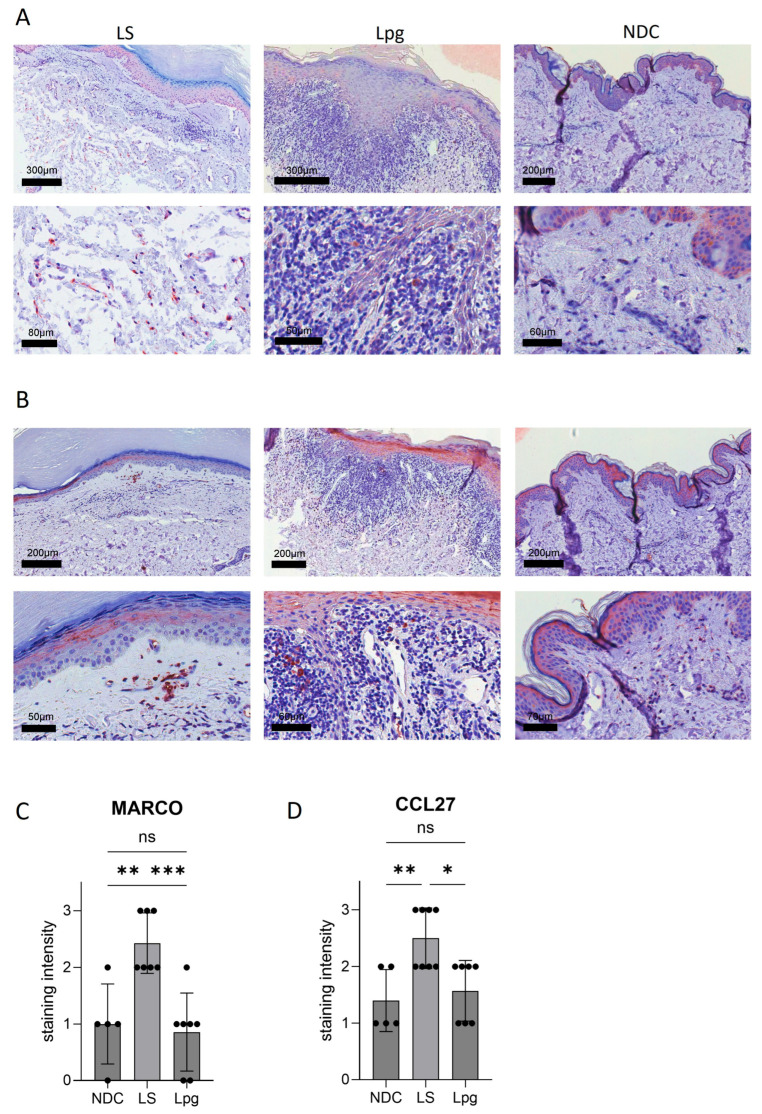
Protein expression of selected genes. Immunohistochemistry stainings with antibodies against *MARCO* (**A**) and *CCL27* (**B**) were performed on lesional skin of LS (*n* = 7) and Lpg (*n* = 7) and of NDC (*n* = 5). Semi-quantification of the staining intensity of *MARCO* (**C**) and *CCL27* (**D**) was performed (scale 0–3). One-way ANOVA was used as statistical test. LS, lichen sclerosus; Lpg, lichen planus genitalis; NDC, non-diseased controls. * *p* < 0.05, ** *p* < 0.01, *** *p* < 0.001, ns: not significant.

While most inflammation-related genes were downregulated in LS compared with Lpg, *CCL27*, coding for a protein involved in T-cell-mediated skin inflammation, was highly upregulated in LS (9.6-fold; *p* = 0.00082; Table 2). Moreover, the Macrophage Receptor With Collagenous Structure (*MARCO*) gene, coding for a protein that is part of the innate antimicrobial immune system, showed much higher expression in LS (5.5-fold; *p* = 0.0022; Table 2). These findings could be confirmed on the protein level by immunohistochemistry stainings (Figure 2A,B).

### 3.4. Higher Abundancy of B Cells in Lpg Compared with LS

**Figure 3:**
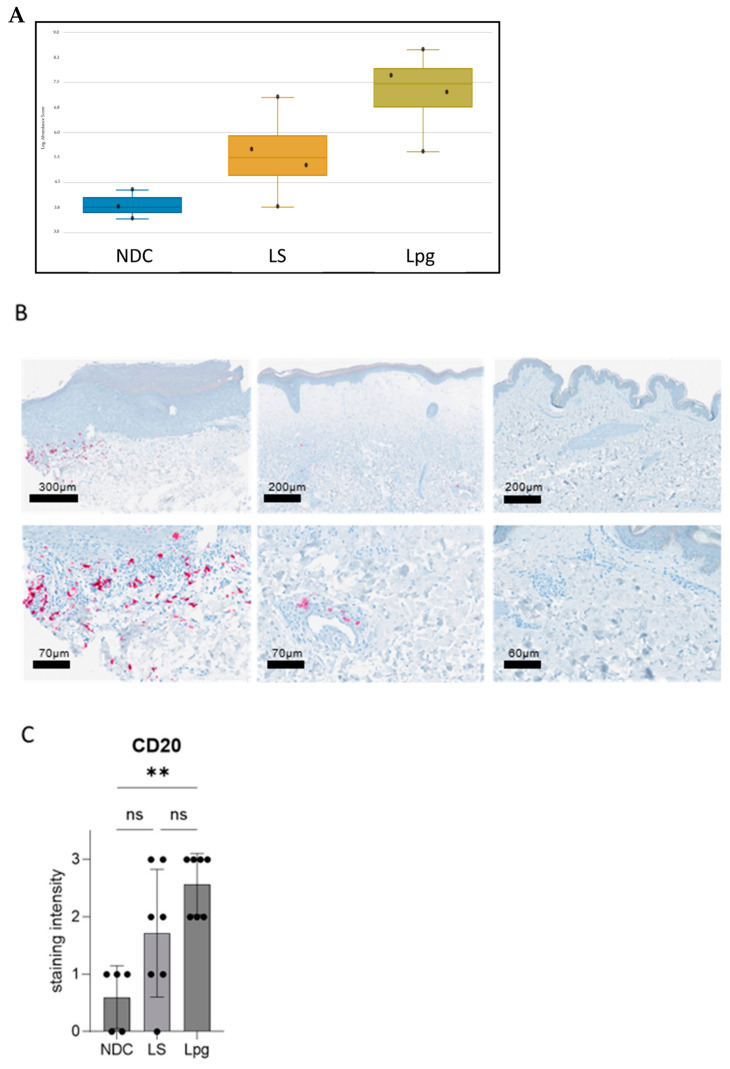
Distinct B-cell expression in lichen sclerosus, genital lichen planus, and healthy controls. Meta-analysis using the databases Cell Atlas and PanglaoDB shows a trend toward upregulation in Lpg compared with LS in the gene expression related to B cells (graphical visualization by Rosalind software) (**A**). Immunohistochemical staining for the B-cell marker CD20 showed higher infiltration of CD20-positive B cells in Lpg lesions (*n* = 7) compared with LS lesional skin (*n* = 7) and NCD (*n* = 5) (**B**). Semi-quantification of the staining intensity of CD20 was performed (scale 0–3). One-way ANOVA was used as statistical test. LS, lichen sclerosus; Lpg, lichen planus genitalis; NDC, non-diseased controls (**C**). ** *p* < 0.01, ns: not significant.

Meta-analysis databases such as Cell Atlas and PanglaoDB were used to study possible differences in the cell type composition between LS, Lpg and NDC according to the expression of defined genes per cell type. While no significant differences between LS and Lpg were detected in other cell types (Supplementary Figure S1A–C), gene expression related to B cells shows a trend toward upregulation in Lpg compared with LS (Figure 3A and Figure S1D). In line with this, there was a significantly higher expression of genes involved in CD22-mediated BCR regulation in Lpg compared with LS (CD79A, CD22, CD79B, and LYN; *p* = 0.04086). Immunohistochemical stainings of B cells using the B-cell marker CD20 confirmed our findings on the protein level, showing a stronger infiltration of CD20-positive cells in Lpg compared with LS lesional skin and NDC (Figure 3B,C).

### 3.5. Interleukin-1 Related-Mediated Signaling Pathway Is Specifically Upregulated in Lpg Compared with LS

A pathway analysis was performed to identify possible key pathways in LS. When compared with NDC, both LS and Lpg showed a significantly higher expression of genes involved in IL-12-mediated signaling events (LS, *p* = 0.006; Lpg, *p* = 0.033). Likewise, genes playing a role in the IL-12/STAT4 pathway were upregulated in both LS and Lpg compared with NDC (LS, *p* = 0.033; Lpg, *p* = 0.052). The direct comparison of LS and Lpg revealed no significant difference in IL-12-mediated signaling events (*p* = 0.549) and in the IL12/STAT4 pathway (*p* = 0.449).

When comparing LS with NDC, a significant association was observed in downstream signaling pathways of naïve CD8^+^ T cells (*p* = 0.005), as well as in TCR signaling (*p* = 0.033). In contrast, comparison of Lpg with NDC showed no significant difference in downstream signaling in naïve CD8^+^ T cells (*p* = 0.063), although TCR signaling remained significantly altered (*p* = 0.016). Direct comparison between LS and Lpg revealed no significant differences in gene expression related to downstream signaling in naïve CD8^+^ T cells (*p* = 0.817), while TCR signaling exhibited a borderline significance (*p* = 0.048) (Table S3 and Table 3).

**Figure 4:**
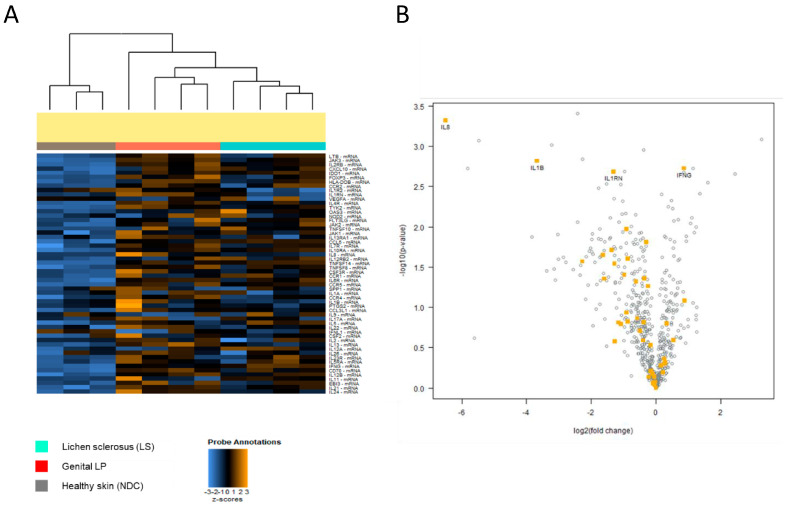
(**A**) Differential expression of IL-1 pathway genes. Distinct expression of IL-1-related genes across Lpg (*n* = 4), LS (*n* = 4), and NDC (*n* = 3) was detected as visualized by heatmap hierarchical clustering. Comparative analysis between LS and Lpg specimens showed strong upregulation of IL1B, IL1RN, IL1R2, and IL6 in Lpg compared with LS lesional skin (**B**).

While genes related to most immune pathways were similarly expressed in both diseases, genes of the IL-1 pathway (IL1B, IL1RN, IL1R2, and IL6) were significantly higher expressed in Lpg compared with LS (*p* = 0.003) (Figure 4A,B, Supplementary Table S4).

## 4. Discussion

This study analyzed clinical and epidemiological data from 174 patients (142 with LS and 32 with Lpg) and compared gene expression profiles of lesional skin of focusing on inflammatory mechanisms.

Clinically, the median age of diagnosis of 62 years of our LS patients was consistent with reports in the literature [19,20]. The increased median age of the LS patients could be attributed to the likelihood that children with LS are typically treated directly in pediatric clinics. In the Lpg cohort, the median age of 53.5 years was slightly higher compared with findings in other studies [10,11]. However, this observation should be interpreted cautiously due to the small sample size. A significant difference in mean age was observed between LS and Lpg patients, with LS patients generally being diagnosed at a later age than Lpg patients, as also reported in the literature [10,21].

Regarding sex distribution, both LS and Lpg patients demonstrated a stronger tendency toward being male compared with previous reports [11,20]. This may be explained by the fact that females are more likely to be treated by their gynecologist, while not all males regularly consult a urologist. In our study, autoimmune diseases were among the most common comorbidities in patients with LS, affecting approximately 31% of the cohort. This finding is consistent with previous studies in the literature [21,22]. Metabolic syndrome-related conditions were the most common comorbidities, with a significant difference in prevalence compared with the general population [23]. Notably, type 2 diabetes mellitus occurred nearly twice as frequently in LS patients compared with the general population. This finding did not reach statistical significance, which may reflect the limited sample size, particularly in the Lpg group (*n* = 32), which reduced statistical power. Nonetheless, a true absence of association between diabetes and LS cannot be excluded. Interestingly, studies have identified associations between LS and oral glucose intolerance, as well as other cardiovascular risk factors [24]. However, a recent study by Liu et al. found no significant differences in the prevalence of diabetes mellitus between individuals with and without LS [25]. Despite this, nutritional interventions, such as low-calorie diets and weight loss, particularly in obese individuals, have been shown to potentially improve inflammatory skin conditions [26]. This suggests that lifestyle modifications could play a role in managing inflammation in LS, even if a direct link between LS and diabetes remains unconfirmed.

Gene expression analysis comparing LS, Lpg, and NDC revealed that LS exhibited higher expression of inflammation-related genes compared with NDC. However, compared with Lpg, LS appeared less immunogenic, with most inflammation-related genes showing downregulation. In detail, the comparison of LS with NDC identified 182 significantly differentially expressed genes, primarily related to cytotoxicity, T-cell function, antigen processing, and NK cell function. Upregulation of CD56+ and CD8+ cell-associated genes in LS aligns with studies showing that lymphocytic infiltrates in LS are predominantly CD8+ T cells activated by Th1 cytokines like IL-12. This activation leads to IFN-γ production, triggering HLA-DR antigen expression in keratinocytes and lesion progression [27]. Afanasyeva et al. link IL-12 activity to the STAT4 pathway, which our pathway analysis confirmed, showing significant upregulation of IL-12/STAT4 signaling. While LS and NDC showed distinct differences, the immune cell composition between LS and Lpg was nearly similar, supporting the shared role of CD8+ cells and IL-12-mediated signaling in both conditions [7,28]. As a result, IL-12 might be a possible therapeutic target for both LS and Lpg. To our knowledge, there are currently no studies about IL12/23 antagonists for LS nor Lpg.

The slight dominance in cytotoxicity score in LS compared with Lpg might be explained by a high proportion of early-stage (active) LS in the study cohort. Early-stage LS has been demonstrated to predominantly show strong cytotoxic gene expression, while more chronic-phase LS presents with fibrosis or immune exhaustion [29]. Therefore, the cytotoxicity score might serve as a disease activity marker of LS. Further, increasing evidence supports LS to be an autoantibody-mediated autoimmune disease with autoreactive T cells [7,30], potentially leading to a pronounced cytotoxic autoimmune phenotype.

When comparing LS with Lpg, we identified 104 significantly differentially expressed immune-related genes, with the majority being downregulated in LS. This suggests that Lpg may exhibit a more immunogenic profile compared with LS. However, several genes were significantly upregulated in LS, with the most prominent being *MARCO* and *CCL27*.

*MARCO* is a scavenger receptor involved in multiple functions, including ligand binding and cell recognition, which are important for macrophage adhesion and migration. It also facilitates phagocytosis and contributes to inflammatory responses in both innate and adaptive immune cells [31]. It was associated with skin inflammation in the context of psoriasis where *MARCO*-expressing macrophages were increased compared with normal skin [32]. To our best knowledge, *MARCO*-expressing cells have not been described in the context of Lichen sclerosus pathogenesis. Given its role in macrophage function and association with skin inflammation in conditions such as psoriasis, *MARCO* may represent a potential diagnostic marker in inflammatory skin diseases like lichen sclerosus.

*CCL27*, along with *MARCO*, was among the most upregulated genes identified in lichen sclerosus. *CCL27* is predominantly expressed in the skin by epidermal keratinocytes and has been linked to skin-resident immune cells such as Langerhans cells via its receptor *CCR10*, facilitating T-cell homing to cutaneous tissues [33]. It attracts cutaneous lymphocyte antigen-positive cells, which express the *CCR10* receptor [34]. Overexpression of *CCL27*, as observed in our findings, enhances the recruitment of *CCR10*^+^ cells, which play a key role in T-cell-mediated skin inflammation [34]. Elevated *CCL27* levels have been reported in the sera of patients with various skin diseases, such as contact dermatitis, psoriasis, and atopic dermatitis, all of which show fluctuating *CCL27* expression [35]. Blocking the *CCR10*/*CCL27* interaction could represent a potential strategy to prevent inflammatory processes in lichen sclerosus. A study by Thangavadivel et al. has already suggested a therapeutic approach using siRNA to inhibit the *CCR10*/*CCL27*/IL-10 pathway in patients with multiple myeloma [36]. Further studies are warranted to explore this pathway, particularly in the context of chronic inflammatory skin diseases.

Notably, four genes involved in CD22-mediated B-cell receptor (BCR) regulation were downregulated in LS compared with Lpg. BCR activation drives antibody production through antigen binding, with CD22 modulating the antibody response by either enhancing or inhibiting it [37]. The downregulation of key B-cell activation genes, such as CD79A and CD79B, in LS suggests a weaker B-cell response compared with Lpg. This aligns with our B-cell analysis, which indicated a greater dominance of B cells in Lpg than in LS, although this difference did not reach statistical significance (*p* = 0.172). In addition, semi-quantification of CD20 immunohistochemistry stainings confirmed a higher abundancy of B cells in Lpg. Reduced activation and lower abundance of B cells in LS compared with Lpg may reflect fundamental differences in the underlying immune response of the two diseases. The dense, interface-dominant lichenoid infiltrate rich in both activated T and B lymphocytes suggests a more pronounced adaptive immune activation. Chronic antigenic stimulation and sustained T-cell activation in Lpg can promote local B-cell recruitment and differentiation through cytokine-mediated crosstalk. This favors the formation of tertiary lymphoid structures and local antibody production within lesional skin or mucosa. Moreover, Lpg has a well-established autoimmune component, and the presence of autoreactive B cells may contribute to epitope spreading and amplification of the inflammatory response. Third, mucosal surfaces—such as the genital epithelium predominantly affected in Lpg—are naturally enriched in lymphoid aggregates and facilitate stronger humoral immune activity compared with the keratinized skin commonly involved in LS.

Altogether, these factors suggest that B cells play a more prominent and possibly earlier role in the immunopathogenesis of Lpg, whereas in LS, their involvement appears secondary and less extensive, likely reflecting later stages of chronic inflammation and fibrosis [6,7,28,32,37].

Interestingly, Lpg exhibited a stronger immunogenic effect through IL-1 signaling compared with LS. Interestingly, Clay et al. found an increased carrier frequency of an intronic copy number variation (CNV) polymorphism of the IL-1 receptor antagonist (IL-1RN) gene in LS patients compared with controls. Moreover, they showed that the frequency of the IL-1RN polymorphism among patients is correlating with LS severity, suggesting that this IL-1RN polymorphism might be a genetic marker for LS severity with potential functional importance [38]. Studies have shown that IL1 plays a role in the pathogenesis of Lichen planus. The study from Qin et al. analyzed CARD18, a caspase recruitment domain, which inhibits caspase-1-mediated activation of the pro-inflammatory cytokine IL-1b. A downregulation in lichen planus was observed, suggesting that IL-1β could have a potential pathogenic role as CARD18 is a negative regulator of inflammasome activation [39]. Notably, a case of a 43-year-old woman with Erdheim–Chester disease and widespread cutaneous lichen planus has been reported who was treated with daily subcutaneous anakinra (100 mg) for her systemic condition. Remarkably, within two days, not only did her Erdheim–Chester disease symptoms improve, but her lichen planus-related pruritus resolved, and many of her lichen planus lesions strongly improved. This suggests that IL-1 may play a role in the inflammatory processes of lichen planus and that anakinra could be a potential therapeutic option, especially in refractory cases [40]. However, this is based on a single case report, and specific studies or documented cases focusing on the use of anakinra for genital lichen planus are lacking.

This study has several limitations. First, its retrospective design introduces the potential for selection and information bias as data collection depended on existing medical records that may be incomplete or inconsistently documented. Second, the unequal cohort sizes between LS and Lpg may affect the robustness of clinical comparisons, while the overall limited sample size—particularly in the NanoString analysis—reduces statistical power and increases the risk of type II errors. Third, as a single-center study conducted at a tertiary referral hospital, referral bias cannot be excluded, and the findings may not fully reflect the clinical spectrum observed in community or primary care settings. Moreover, the predominance of Caucasian patients further limits the generalizability of our results to more diverse populations. Finally, the observational and cross-sectional nature of the study precludes conclusions regarding causality between clinical or molecular parameters and disease mechanisms. Future prospective, multicenter studies with larger and ethnically diverse cohorts are warranted to validate and expand upon these findings.

Nevertheless, the study provides a comprehensive comparative analysis of LS, Lpg, and NDC at both epidemiological and molecular levels, based on real-world data collected over a ten-year period. By identifying differentially upregulated genes, it highlights key inflammatory pathways potentially involved in pathogenesis and offers valuable insights into future therapeutic strategies. In line with previous research, the study confirmed IL-12 pathway upregulation in both LS and Lpg, supporting IL-12/23 antagonists as a potential shared treatment. Notably, it also revealed a prominent role of IL-1-driven inflammation in Lpg, suggesting IL-1-targeting agents such as anakinra as promising therapeutic options. To validate and expand upon these findings, future multicenter studies involving larger and more diverse cohorts, along with focused mechanistic investigations, are warranted.

## Figures and Tables

**Table 1:** Clinical features of LS compared with Lpg.

	LS	Lpg	*p*-Value
**Number of patients**	142	32	
**Age/median**	14–93/62	20–78/53	0.00386
**Sex**			
Female	78 (55%)	10 (31%)	0.01550
Male	64 (45%)	22 (69%)	0.01550
**Type of LS**			
Genital only	125 (88%)	17 (53%)	0.00001
Genital + extragenital	17 (12%)	15 (47%)	0.00001
**Comorbidities**			
Metabolic syndrome	71 (50%)	7 (22%)	0.03852
Hypertension	37 (26%)	3 (9%)	0.04276
Diabetes mellitus type 2	18 (13%)	3 (9%)	0.60457
Dyslipoproteinemia	14 (10%)	1 (3%)	0.22667
Autoimmune diseases	44 (31%)	9 (28%)	0.67583
Hepatitis B/C infection	7 (5%)	2 (7%)	0.76061

**Table 2:** Top 18 upregulated genes in LS compared with Lpg.

Gene Name	Fold Change	*p*-Value
*CCL27*	9.55125	0.00082
*MARCO*	5.4807	0.00220
*CFD*	3.05171	0.00281
*CXCL14*	2.57319	0.00386
*GZMH*	2.35832	0.01011
*MST1R*	2.2628	0.01177
*CREB5*	2.20201	0.00190
*C6*	2.16129	0.00775
*NT5E*	2.10345	0.00904
*C2*	1.91899	0.01316
*SIGLEC1*	1.91526	0.01515
*IFNG*	1.83616	0.00189
*XCL2*	1.80402	0.00209
*ITGA2*	1.6683	0.04843
*CD160*	1.65957	0.02528
*HLA-G*	1.62132	0.00811
*ITGAM*	1.6146	0.02613
*MRC1*	1.5971	0.04956

**Table 3:** Important in LS compared with Lpg (Gene Set Analysis Significance Scores).

Term Name	Directed Global Significance Score	Global Significance Score	# of Genes in Term
Cytotoxicity	1.7082	1.9881	10
Complement	1.643	1.7624	15
Antigen processing	1.0967	1.2809	22
Transporter functions	1.0753	1.7324	22
Senescence	−0.3817	1.1772	12
NK cell functions	−0.6734	1.9272	31
Macrophage functions	−0.7495	2.1201	15
Cell functions	−1.0654	1.8345	70
Leukocyte functions	−1.1671	2.588	8
Microglia functions	−1.1867	2.0073	5
T-cell functions	−1.2103	1.7363	70
Adhesion	−1.2587	2.0096	25
Cell cycle	−1.3787	1.5133	13
CT antigen	−1.4201	1.9419	27
Regulation	−1.4338	2.0148	155
Chemokines	−1.4812	2.1429	99
TNF superfamily	−1.6006	1.8693	30
TLR	−1.7065	2.1575	11

## Data Availability

The datasets generated and analyzed during the current study are not publicly available due to privacy and ethical considerations. However, they are available from the corresponding author upon reasonable request.

## Supplementary Materials

The following supporting information can be downloaded at https://www.mdpi.com/article/10.3390/biomedicines13112817/s1: Table S1. Top 20 upregulated genes in LS compared with NDC; Table S2. Top seven downregulated genes in LS compared with NDC; Table S3. Important biological functions in LS compared with NDC (Gene Set Analysis Significance Scores); Table S4. Top 20 downregulated genes in LS compared with Lpg; Figure S1. Molecular separation of genes associated with different cell types. Heatmap visualization shows hierarchical clustering of genes associated with macrophages (A), NK cells (B), T-cell function (C), and B cells (D) in Lpg (*n* = 4), LS (*n* = 4), and NDC (*n* = 3) samples. LS, lichen sclerosus; Lpg, Lichen planus genitalis; NDC, non-diseased controls.

## Abbreviations

The following abbreviations are used in this manuscript:

LS	Lichen sclerosus
Lpg	Lichen planus genitalis
LP	Lichen planus
GC	Glucocorticoids
NDC	Non-diseased controls
FFPE	Formalin-fixed, paraffin-embedded
BSA	Bovine serum albumin
PBS	Phosphate-buffered saline
AEC	Aminoethyl carbazole
RNA	Ribonucleic acid
IL	Interleukin
IFN-γ	Interferon gamma
TNF	Tumor necrosis factor
TLR	Toll-like receptor
NK	Natural killer cell
BCR	B-cell receptor
HBV	Hepatitis B virus
HCV	Hepatitis C virus
