# Supplementary material for: Notable Differences in Clinical Features and Inflammatory Gene Expression Between Genital Lichen Sclerosus and Lichen Planus
Source: Biomedicines. 2025 Nov 19;13(11):2817. doi: 10.3390/biomedicines13112817 (PMC12650408; doi:10.3390/biomedicines13112817)
Supplement: Supplementary file 1 [file biomedicines-13-02817-s001.zip › biomedicines-3944292-supplementary.pdf]

Supplementary Table S1. Top 20 upregulated genes in LS compared to NDC

Gene name	Fold change	p-value
BAGE	73.4999	0.00111
CXCL9	46.1452	0.00125
MS4A1	45.4686	0.00666
CD8A	33.5095	0.00060
GZMB	25.461	0.00020
CD160	23.4001	0.00137
CXCL11	22.0	0.00095
NEFL	20.025	0.00332
CXCL13	19.2428	0.00153
CCL5	18.9781	0.00052
PDCD1	18.675	0.00282
PMCH	17.85	0.00691
GZMK	17.0565	0.00276
GZMH	16.0691	0.00044
KLRK1	15.3287	0.00058
KLRD1	14.97	0.00023
C4B	14.7177	0.00350
TBX21	14.5367	0.00029
EOMES	14.0454	0.00220
TNFRSF9	13.6811	0.00029

Supplementary Table S2. Top 7 downregulated genes in LS compared to NDC

Gene name	Fold change	p-value
TMEFF2	-6.66669	0.00754
HSD11B1	-6.50816	0.00078
RORC	-5.33334	0.00173
ALCAM	-3.43119	0.00029
HMGB1	-3.1613	4.22e-5
GATA3	-2.95679	0.00345
IL34	-2.83657	0.00915

Supplementary Table S3. Important biological functions in LS compared to NDC (Gene Set Analysis Significance Scores)

Term Name	Directed Global Significance Score	Global Significance Score	# of Genes
Cytotoxicity	9.0178	9.0178	10
NK Cell Functions	6.5298	6.5298	31
Antigen Processing	6.3623	6.39	22
T-Cell Functions	5.7315	5.8125	70
Pathogen Defense	5.1868	5.2616	12
TNF Superfamily	4.7681	4.7892	30
Leukocyte Functions	4.5846	4.5891	8
Regulation	4.5671	5.1708	155
Cytokines	4.5388	4.5941	56
B-Cell Functions	4.3499	4.3499	25

TLR	4.2451	4.2457	11
Complement	4.1932	4.3646	15
Interleukins	4.1679	4.3797	38
Chemokines	4.0681	4.2422	99
Macrophage Functions	3.8033	4.2334	15

Supplementary Table S4. Top 20 downregulated genes in LS compared to Lpg

Gene name	Fold change	p-value
CXCL8	-90.286	0.00048
CXCL6	-56.671	0.00190
CEACAM6	-44.4444	0.00086
TREM1	-14.2457	0.01334
IL1B	-12.6985	0.00153
S100A12	-10.3672	0.03522
CXCL1	-9.33869	0.00097
IL6	-8.86487	0.03342
LCN2	-8.71711	0.04602
FPR2	-8.22411	0.01254
CD79A	-8.05916	0.02512
CEACAM1	-7.26413	0.02399
LTF	-6.93103	0.02770
TNFRSF17	-5.88271	0.03400
MME	-5.36363	0.00040
LILRA5	-5.35512	0.00877
CR2	-5.09676	0.03966
MUC1	-5.00212	0.03029
AIRE	-4.86956	0.00146
PTGS2	-4.81034	0.02697

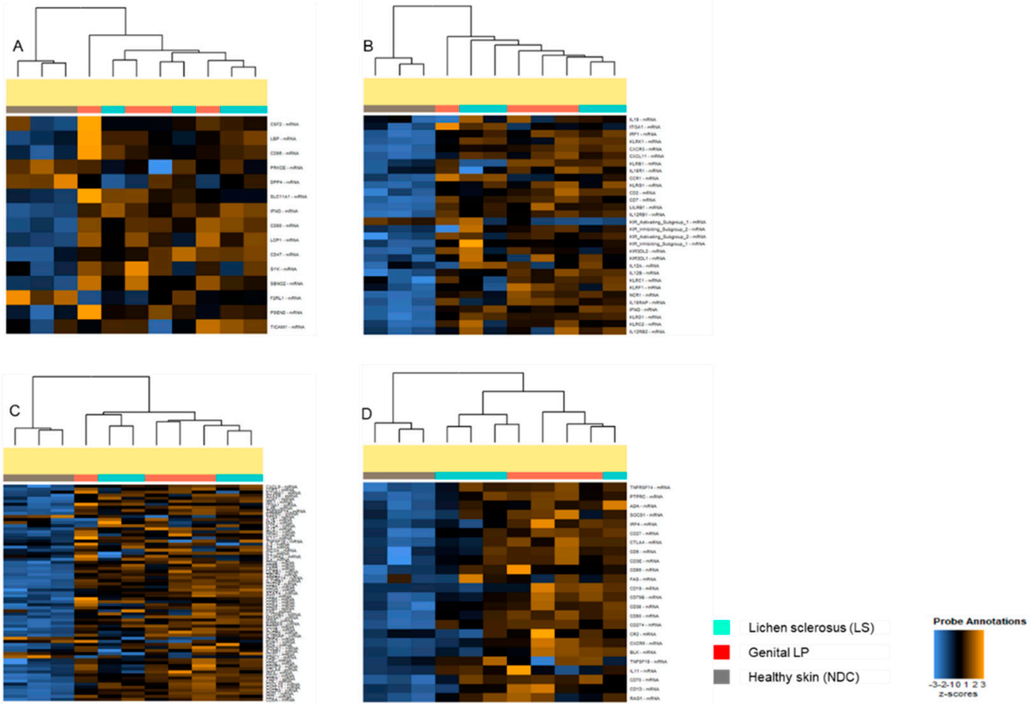

Supplementary Figure S1. Molecular separation of genes associated with different cell types. Heatmap visualization shows hierarchical clustering of genes associated with macrophages (A), NK cells (B), T cell function (C), and B cells (D) in Lpg (n=4), LS (n=4), and NDC (n=3) samples. LS: lichen sclerosus; Lpg: Lichen planus genitalis; NDC: non-diseased controls.
